# Security Risk Measurement for Information Leakage in IoT-Based Smart Homes from a Situational Awareness Perspective

**DOI:** 10.3390/s19092148

**Published:** 2019-05-09

**Authors:** Mookyu Park, Haengrok Oh, Kyungho Lee

**Affiliations:** 1 School of Information Security, Korea University, Seoul 02841, Korea; ctupmk@korea.ac.kr; 2 Agency for Defense Development (ADD), Seoul 05771, Korea; haengrok@add.re.kr

**Keywords:** smart homes, Internet of Things (IoT), risk measurement, cyber-situational awareness

## Abstract

Internet-of-Things (IoT) is a technology that is extensively being used in various fields. Companies like Samsung, LG, and Apple are launching home appliances that use IoT as a part of their smart home business. Currently, Intelligent Things which combine artificial intelligence (AI) and IoT are being developed. Most of these devices are configured to collect and respond to human behavior (motion, voice, etc.) through built-in sensors. If IoT devices do not ensure high security, personal information could be leaked. This paper describes the IoT security threats that can cause information leakage from a hierarchical viewpoint of cyberspace. In addition, because these smart home-based IoT devices are closely related to human life, considering social damage is a problem. To overcome this, we propose a framework to measure the risk of IoT devices based on security scenarios that can occur in a smart home.

## 1. Introduction

A smart home consists of a computer, smart phone, and other devices that are equipped with an Internet-of-things (IoT) connection. In recent years, the use of artificial intelligene (AI)-integrated intelligent IoT devices has significantly increased [1]. For example, a smart sensor, such as a thermostat, can be remotely controlled by a user via an Internet connection. The user can monitor the house in real-time through the IP camera. Even door locks that include connectivity options allowing remote operation are being developed. This phenomenon increases the possibility of threats to the real space, unlike cyberattacks (DDoS, APT attacks, etc.), which cause damage to the cyberspace. A smart home is vulnerable to security threats because it uses the Internet that utilizes Radio Frequency Identification (RFID), Wireless Sensor Network (WSN), Wi-Fi, 3G, and 4G. This implies that the information collected by the sensors installed in the IoT devices can leak personal information to the attacker owing to their vulnerability.

These cyberattacks focus on the collection and monitoring of information. In 2014, e-mail leaks confirmed that many countries’ intelligence and investigation agencies had requested an Italian hacking team to create an exploit kit that exploited vulnerabilities in IoT equipment for surveillance or inspection purposes. In 2017, Vault 7, the primary publication of confidential documents, leaked information about the activities of intelligence agencies by WikiLeaks, an international non-profit organization that publishes classified information. This document contained information on a cyber weapon developed by the CIA in the United States and GCHQ in the United Kingdom. It can monitor users by exploiting vulnerabilities through Samsung’s smart TV. In addition, the disclosed document explains techniques to acquire the root privilege of a smartphone by exploiting vulnerabilities of browsers such as Chrome, Opera, and Samsung mobile browser. It is evident from these instances that IoT device sensors can be used as an information collection path by an intruder [2].

The IoT environment is advantageous because it configures the user’s surrounding as a connected environment to provide convenience. However, because most IoT devices are connected to the Internet, their security can be challenged by a single vulnerability. A malicious attacker may steal confidential information stored on IoT devices, monitor the user’s life, or, if necessary, the user’s personal information may be unauthorisedly used. Therefore, in the development of IoT devices, risk identification and measurement and assessment procedures should be introduced to identify the countermeasures against unauthorized user identification and access control. This paper addresses the threats that may occur owing to IoT device sensors by:

- Comparing and analyzing the cyberspace layers and IoT environment.
- Connecting with the cyberspace layer to identify possible threats to IoT devices in a smart home.
- Proposing a framework for risk assessment of IoT devices in a smart home environment.

This study explains security threats such as privacy infringement and personal information leak in smart homes composed of IoT devices. It approaches the evaluation method of these threats from the framework of situational awareness (SA) and presents the direction of IoT threat perception from the viewpoint of decision-makers or managers.

## 2. Scalability of Cyber Threats

In the past, cyberattacks caused one-dimensional damage, which caused service failure through vulnerabilities such as hardware and software. However, recent cyberattacks are causing harm to the psychological and financial states of human beings with the aim of unauthorised acquisition of information. With the developments in IoT technology, there is an increase in the number of threats related to an individual’s privacy breach. This section describes the relationship between the IoT environment and cyber threats.

### 2.1. IoT Layered Architecture and Cyber Threat

There is no general architecture for IoT environments. However, the typical IoT layered architecture, described in terms of security, consists of the perception layer, network layer, processing layer, application layer, and business layer [3].

- Perception Layer: This layer, also called the sensor layer, is responsible for the identification of objects and collection of information about objects. RFID, 2-D barcodes, and various types of sensors for object recognition are attached to the things. The information collected by these sensors varies in location, atmosphere, environment, motion, and vibration. These sensors can be used as a tool to unauthorisedly monitor privacy by an attacker.
- Network Layer: This layer connects the perception layer and application layer. In other words, this layer is responsible for the transfer of data collected in the perception layer to other connected devices through a communication channel. The transmission medium may be wired or wireless (Wi-Fi, Bluetooth, Zigbee, cellular network, etc.). The connectivity of IoT devices is vulnerable to transfer malware and network attacks such as denial of service.
- Processing Layer: This layer collects and processes the information transmitted from the network layer. It is responsible for removing meaningless extra information and extracting useful information. This layer can affect the performance of the IoT when a large amount of information is received.
- Application Layer: This layer uses IoT technology or defines all applications implemented to IoT. IoT can be implemented in smart homes, smart cities, and smartphones, which controls them. Because the services provided depend on the information collected by the sensors, they may be different for each application. Especially when IoT is used in a smart home, various internal and external threats and vulnerabilities can occur.
- Business Layer: The business layer represents the intended behavior of the application. This layer is responsible for managing and controlling the application, business, and revenue models of IoT and manages user’s personal information. This layer of vulnerability could allow an attacker to misuse the application.

IoT environments such as smart homes are remotely controlled by mobile devices such as smartphones. From the viewpoint of IoT layered architecture, smartphone sensors can cause secondary damage such as personal information leakage. If an application is installed in this device, the damage can be extended.

### 2.2. Connection between Cyberspace and Real World

Cyberspace is a specific space that can overcome the temporal and spatial limitations of the real world by utilizing the virtual environment formed with electronic devices and electronic spectrum, and enables communication through it. Cyberspace is characterized by the ability to overcome geographical limitations and provide rapid delivery of extensive information. However, as the usability of cyberspaces increases, the components of cyberspace such as the network, embedded systems, and internet, and real-world components such as humans, society, and policy, are changing interdependently.

According to the “Joint Publication (JP) 3-12R Cyber Operation” published by the US Chief of Staff, the concept of abstract cyberspace is described in a hierarchical structure. According to this publication, cyberspace consists of three layers: physical, logical, and persona. The physical layer is a hierarchy in which physical devices (routers, switches, sensors, etc.) are located geographically or physically. The logical layer is a layer that logically connects devices located in the physical layer through software or protocols. Representative layers included in this layer are the seven layer of Open Systems Interconnection (OSI). The persona layer is a layer that forms or recognizes a human in the cyberspace through the electronic service formed by the physical and logical layers. For example, IP address, E-mail, and ID are forms of persona layer. The threat of cyberspace is projected into the real world through this persona layer. This hierarchical concept of cyberspace can be utilized as a framework to identify cyber threats and damage in IoT equipment [4].

**Figure 1:**
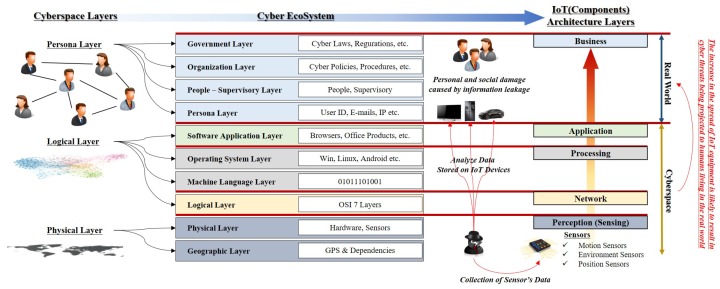
Owing to developments in technology, cyberspace has become connected to real world and the result of threat in cyberspace can be extended to real world.

In a report published in 2011, the US Department of Homeland Security presented a cyber ecosystem that explains the concept of cyberspace layers. The cyber ecosystem is a concept that constitutes a circulating virtual ecosystem by interacting with people, governments, social organizations, policies, laws, and electronic devices such as hardware and software. It consists of fifteen layers with complex interactions. The top four layers (persona, people-supervisory, organization, government layer), in particular, conceptually explained that the outcome of threats in cyberspace may ultimately affect individuals or society [5]. This concept can be explained in connection with the components of IoT (See Figure 1).

- Perception (Sensing)—Physical or Geographic Layer: The main purpose of *Perception* is to identify the phenomenon and change in the device environment based on various sensors and consequently collect data from the real world. These motion, environment, and position sensors are physically located and can be connected to the physical layer. The position sensors are also used as a tool to measure geographical locations.
- Network—Logical Layer: Sensors in IoT devices are integrated through a sensor hub, which uses transport mechanisms such as Inter-Integrated Circuit (I2C) or Serial Peripheral Interface (SPI) to ensure data flow between sensors and applications. The network component of IoT is responsible for transferring the data collected through sensors to other connected devices. These components are similar in concept to the logical layer because they logically connect nodes in cyberspace. Various technologies such as Wi-Fi, Bluetooth, and Zigbee are used to connect data flows between different devices.
- Processing—Machine Language or OS Layer: Processing component consists of the main data processing unit of an IoT device. This configuration corresponds to the machine language layer. In addition, processing performs the decision-making analysis through the data collected from sensors. IoT devices such as smart home hubs serve to improve the user experience by storing the analysis results. Because the OS is essential for the execution of this analysis, it can be connected to the OS layer.
- Application—Application Layer: The application component is responsible for implementing and presenting the results of data processing to perform different applications through IoT devices. The application is a user-oriented layer that performs various tasks for the user and serves as the application layer of the cyber ecosystem. IoT devices can be implemented in smart homes, personal hygiene, and healthcare.
- Business—Real World: A business represents the intended behavior of an application and is closely related to the real world in terms of managing the user’s personal information. However, it is difficult to provide proper security through a sensor management system and security system, which have strong software tendency. The sensor data captured by a malicious attacker may be processed and cause problems such as leakage of personal information and unauthorised privacy monitoring.

## 3. Risk Assessment Approach Using Factor Analysis of Information Risk (FAIR)

Risk analysis and measurement consist of setting the scope of assets, identification of threats and vulnerabilities, and analysis of threat agents (TCom, Threat Community) based on various scenarios. Risk assessment is divided into basic risk assessment and detailed risk assessment. Although basic risk assessment does not utilize an engine to calculate the risk level, it can produce results quickly and easily. However, it has difficulties in verifying the effectiveness of countermeasures. A detailed risk assessment has the advantage of being highly reliable in risk measurement and it is capable of aggressive countermeasures; however, its qualitative measurement is unreliable. To overcome the shortcomings of this risk assessment, this study implemented the Factor Analysis of Information Risk (FAIR). The FAIR method is a risk measurement method developed by J.A. Jones. Unlike the existing qualitative risk measurement model, the FAIR method stochastically approaches the measurement of each factor against assets and threats.

**Figure 2:**
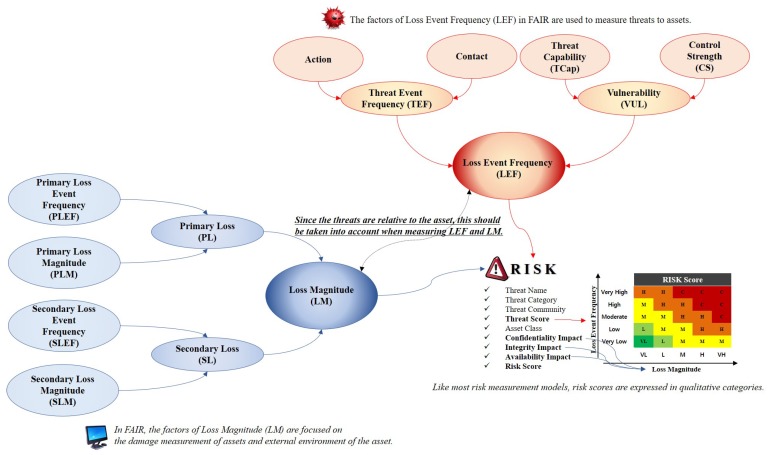
FAIR model is used to measure risk based on likelihood and probability, and consists of *LEF* and *LM* factors that represent threats and damage to assets, respectively.

Because risk is an uncertain event in the FAIR method, the analysis primarily focuses on the likelihood of a set scenario. The process of analyzing risks using FAIR consists of characterizing the risks, combining the risk factors, and measuring the risks. To accomplish this, we approached each factor from a stochastic point of view and classified risks into frequency and magnitude. The FAIR method is divided into loss event frequency (LEF), which is the frequency of the threat, and loss magnitude (LM), which represents the degree of damage to the asset (see Figure 2) [6,7].

**Loss Event Frequency (LEF):***LEF* is the frequency with which the threat agent is likely to damage the acquired assets within a certain period of time. It is essential that the *LEF* distinguishes between the possibilities and probabilities over time to identify threats. This factor measures the attacker’s threat by *threat event frequency (TEF)* and *vulnerability (VUL)*.

- **Threat Event Frequency (TEF):***TEF* indicates the frequency with which a threat agent is likely to act on an asset within a specified period. Similar to the definition of *LEF*, this factor does not reflect the success of an attack by a threat agent. The *TEF* is measured by contact and action factors, where the action against the attack is based on the contact by the threat agent. *Contact* factor means the frequency with which a threat agent is likely to contact an asset within a certain period, and is classified into random, regular, and intentional. The factors that determine the probability of this contact factor are the size of the threat, number of threats in contact, size of the asset, activity of the threat, flexibility of the threat, and relationship between the threat and asset. *Action* refers to the probability that an actual attack will be carried out on an asset that it owns in the event of a threat agent’s contact. The precondition for the action is that the threat agent who can think of is caused by a threat agent intentionally created such as a malicious program. Measures related to these actions include asset value, level of effort, and the probability of detecting threats and experiencing unacceptable consequences.
- **Vulnerability (VUL):***VUL* is the probability that the acquired assets cannot resist the threat agent’s behavior. Vulnerability exists owing to the difference between the capability of the threat agent and that of the asset to resist the capability. This implies that vulnerability is relative to the attack method or type of threat. These *VUL*s are calculated as a combination of *threat capability (TCap)* and *control strength (CS)*. *TCap* is the ability of the threat agent to negatively affect the asset. *TCap* does not equally generate all threats. Therefore, the threat agent does not perform the same functions. In addition, the value of *TCap* may be higher for an attack target where a threat agent is set but it may be incompetent for other objects. *CS* is the resistance strength of the acquired assets when compared to the measured threats. These *CS*s are divided into three types: policies, processes, and techniques. When a small number of controls are set, the probability values of each control can be independently calculated.

**Loss Magnitude (LM):** Loss Magnitude (LM): *LM* is the amount of damage that an attacking target receives when a threat occurs. However, in the case of asset damage, there is no standard framework for asset loss because most organizations or agencies focus on the threat response. To compensate for these weaknesses, the FAIR model combines *primary loss (PL)* and *secondary loss (SL)* to measure the damage.

- **Primary Loss (PL):***PL* consists of *primary loss event frequency (PLEF)* and *primary loss magnitude (PLM)*. *PLEF* is an element of actions that a threat agent performs on an asset and consists of access, misuse, disclose, modify, and deny access. The intent of the threat agent is largely determined by the motivation (e.g., financial gains, revenge, etc.) and nature of the asset, and the magnitude of the loss depends on the location of the threat agent (outside or inside the organization). *PLEF* broadcasts threat competence, which is a characteristic that can cause damage. *PLM* consists of a combination of criticality, cost, and sensitivity. Criticality refers to the nature of assets that causes an impact on the productivity of the organization. Cost indicates the cost of responding to an asset when it is damaged by an attack. Sensitivity refers to the degree of damage that can result from unintended exposure and reflects qualitative measurements such as reputation, competitive advantage, and legal/regulatory proceedings.
- **Secondary Loss (SL):***SL* is the loss caused by the external characteristics of an environment when a threat event occurs. *SL* is measured by a combination of *secondary loss event frequency (SLEF)* and *secondary loss magnitude (SLM)*. *SLEF* is measured considering the timing, due diligence, response, and detection. Threat events result in a direct loss owing to the importance of the asset and inherent value characteristics but *SLEF* is based on changes in the external environment. *SLM* reflects changes in the legal and regulatory landscape, competitive landscape, media, and external stakeholder. When cyberattacks lead to social disruption, various indicators of each factor are reflected.

Complexity can occur in risk decision making in situations that directly reflect human life in the IoT environment. This paper used the framework of the FAIR model, which can measure secondary damage under the assumption that cyber threats damage the real world apart from the cyberspace.

## 4. Risk Assessment and Situation Awareness in IoT Environment

As the availability of IoT technologies increases, it is essential to measure its related threats and risks. Especially, IoT environment, which has high connectivity with people such as smart homes and smart offices, is associated with privacy exposure, which adversely affects the threat. The complexity of this IoT environment makes it difficult for decision-makers to make an accurate assessment of the situation. This section describes the research on risk assessment and SA in the IoT environment.

### 4.1. Research on Risk Assessment in the IoT Environment

The application of IoT technologies poses privacy concerns considering confidentiality, reliability, and integrity of the data that IoT objects interact with. To identify these problems, a research has been conducted to identify the risks. M. Burhan et al. outlined a layered architecture of IoT elements and attacks related to security in this layer. Through this research, this study proposed a new security layered architecture of IoT [3]. B. Ali and A.I. Awad conducted a study using OCTAVE Allegro, an operationally critical threat, asset, and vulnerability evaluation (OCTAVE) methodology to evaluate the security risks of a smart home. This research identifies the security vulnerabilities in smart home IoT devices and suggests ways to reduce those identified risks [8]. C. Liu et al. proposed a dynamic risk assessment method for IoT using an artificial immune system to dynamically evaluate the risk of IoT devices and accordingly determine the situation. This method consists of attack detection agent and a dynamic risk assessment subsystem. Through this method, this research deduced the immune principle and mechanism for risk in IoT environment as a set theory of mathematics [9].

S. Sicari et al. proposed a methodology for the implementation of end-to-end systems to general-purpose methodologies to asses risks to determine the stability and robustness of malicious attacks on the components of the IoT platform. The proposed approach assessed risk by objectively considering the static and dynamic features and components of the IoT system in accordance with the life cycle of data [10]. P.K. Chhouhan et al. proposed a situational assessment approach to identify security risks in IoT devices. This research presented the basic elements of the IoT model and conducted a situational assessment of IoT applications. As a result, this research have addressed the need for increased security for local, transport, and data stores as a countermeasure against the risks [11]. V.L. Shivraj et al. proposed a model-based risk assessment framework based on graph theory to solve the complexity of IoT technologies such as communication protocols, devices, and environments. This research conducted a risk assessment using attack propagation through the bipartite graph technique [12].

A.W. Atamli and A. Martin conducted an assessment of the threat model based on use cases related to IoT. This model classifies threat agents and attack vectors for power management, smart car, and smart heathcare system, and measures the impact of risk on them. These findings can contribute to identifying security requirements to protect each system and ensuring the privacy of users [13]. T. Wu and G. Zhao proposed a risk assessment model that derives the weights of the probabilistic causal relationships of the evaluation factors and the influence relationships of the propagation path in order to solve the personal information security problem of IoT application. This model uses the decision-making trial and evaluation laboratory according to the Bayesian network structure to easily identify the relevant risk propagation path and calculate the weight of each path. Through this research, this research has determined the risk level of assets and each risk propagation path and contributed to the rationale for decision making on privacy security risk assessment [14].

J.R.C. Nurse et al. noted the need for a model that considers the dynamics and originality of IoT based on existing risk assessment models. The researchers proposed a risk assessment model with the view that the risk of cyberspace can be projected into the real world through a combination of current high levels of connectivity or digital, cyber-physical, and social systems [15]. R. Heartfield et al. classifies applicable cyber threats for smart home according to taxonomy and measures not only the attack vector, but also the potential impact of the system on the user. This research classifies the twenty five smart home attacks using the proposed method and draws the results of the legitimate but vulnerable smart home configuration that can lead to the second attack path [16]. K. Ghirardello et al. presented a smart home reference architecture for security analysis. This model identifies the various functions and components of IoT devices and networks installed in a smart home from three viewpoints: functional, physical, and communication. This research proposed a method to utilize the architecture by applying the attack surface to the home automation environment to determine the major vulnerabilities [17].

M. Vitunskaite et al. evaluated smart cities and cybersecurity measures to address the limitations of security requirements for environments with IoT technology. This research proposed a security recommendation framework by comparing management models, security measures, and technical standards for smart cities in Barcelona, Singapore, and London [18]. I. Butun et al. conducted vulnerability identification and risk assessment related to a low-power wide-area network (LPWAN) to implement IoT-based applications. This research analyzed the comprehensive security risks of the protocol and suggested a countermeasure to reduce these risks. In particular, the researchers identified important real threats such as end-device physical capture, rogue gateways, and self-replay [19].

These researches, unlike existing security risk assessment, attempted to solve the complexity of IoT technology. However, there is a limit to determine a risk assessment method that can judge a comprehensive threat situation as the decision-maker or manager. To solve this problem, this study utilizes SA, which is a core concept of the command and control system of the military sector.

### 4.2. SA in Cyberspace

IoT enabled devices provide services to users based on the data collected through various sensors. For example, data collected from IoT devices in a smart home is personal information such as a user’s life pattern, eating habits, and health, among others. However, data leak due to the vulnerabilities in IoT devices can lead to privacy information leak, resulting in incidental damage. The concept of SA is essential for considering incidental damage. SA refers to the process of collecting, understanding, and projecting various environmental factors and data related to time and space when a specific event occurs, and projecting it to future situations. In general, SA aims at understanding the operating environment and status of related organizations to support optimized decision-making in complex and dynamic environments. Typically, a SA model is used in air traffic control, power plant operation, emergency medical services, military’s command and control systems, among others [20].

SA models are proposed and developed based on Endsley’s model proposed by M. Endsley. It consists of *perception* (*level 1*), *comprehension* (*level 2*), and *projection* (*level 3*) to determine the situation. *Perception* (*level 1*) is the level that recognizes the status and attributes of the related element in the environment where the event occurs. This level recognizes the status, attributes, and dynamics of related elements in a given environment. *Comprehension* (*level 2*) is a level that synthesizes the separated perceptual elements through the analysis and evaluation process based on the data collected in *level 1*. *Level 2* helps to understand the impact on the user’s goals to have a comprehensive understanding of the environment. *Projection* (*level 3*) is a level that predicts the effect of analyzing the information obtained in *Level 2* on the state of future operating environment. This level can project an element’s future action in the current environment. This model is designed to prioritize the recognition of the state of system and user elements. The system element is intended to recognize the current state of the asset and enhance the effectiveness of decision-making by making future projections. However, the user factor influences final decision-making by individual ability and experience; thus, a quantitative measure of risk is required [21].

**Figure 3:**
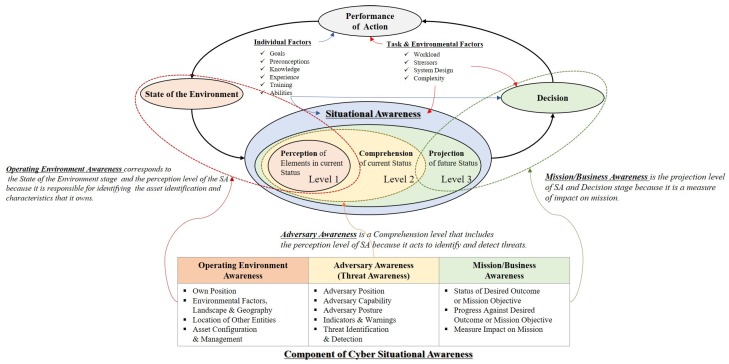
The SA model is being developed based on Endsley’s model. Cyber SA consists of *Operating Environment Awareness*, *Adversary Awareness*, and *Mission/Business Awareness*, and each component are combined with Endsley’s model levels.

SA based on Endsley’s model is being developed to suit the characteristics and purpose of cyberspace. Cyber SA considers the sociotechnical system of systems (STSOS), which focuses on scalability based on the concept of a cyberspace layer. STSOS refers to complex systems with connection threads and information fabrics that enable and shape modern societies, cultures, economies, technologies, and industries. Thus, to achieve cyber SA, the low-level details should be summarized and reflected in the organization’s mission or business perspective. Comprehensive cyber SA consists of computing and network components, threat intelligence, and mission awareness. *Operating environment awareness* aims to organize and manage defined assets. *Adversary awareness* identifies and tracks suspicious actions and integrates knowledge of external and internal threats. *Mission/business awareness* aims to understand the dependencies between elements to identify the threats and the impact of their assets on their mission [22] (see Figure 3).

Based on this comprehensive concept, cyber SA is being studied and developed as a suitable model for data analysis. J. Okolica et al. proposed an automated Cyber SA Model (CSAM) that real-time updates the system environment to reflect the business continuity planning [23]. G.P. Tadda and J.S. Salerno proposed a SA Reference Model (SARM) applying a data fusion model (DFM) to estimate and evaluate the environment under observation based on the collected data. This SARM utilizes data analysis and has the advantage of being able to respond to threats in real time [24]. N. Evancich et al. proposed Effective Cyber SA (ECSA) focusing on network threats. ECSA is composed of *Network Awareness*, *Threat Awareness*, and *Operational Awareness* to comply with the comprehensive concept of cyber SA. *Network Awareness* is the step of collecting data by recognizing the ownership, security characteristics, vulnerabilities, etc. of the network that you have. *Threat Awareness* is intended to detect attack vectors that can be a threat to your network assets. *Operational Awareness* is a measure of the impact that an attack can have on operational capability [25]. J. Webb et al. proposed a situation-aware ISRM (SA-ISRM) model to supplement erroneous decisions and inappropriate security strategies in the information security risk management (ISRM) process. This model has resulted in an enterprise-wide collection, analysis, and reporting of risk-related information that can resolve existing risk assessment flaws in cyberspace [26].

This SA is also applied to the IoT environment. F.G. Toro & A. Tsourdos proposed an augmented reality (AR) tool for the SA of unmanned aerial vehicle (UAV) in an IoT environment. This research utilized the SA on the ground control system (GCS) used by pilots on traditional aerial platforms while converging information on video streams, mission plans, and information from other sensors [27]. P. Vanveerdeghem et al. proposed a SA model for wearable wireless body sensor networks. This research implemented a decision support system by applying SA to the comprehensive representation of sensor data collected from the body [28]. E.G. Zimbelman et al. proposed a SA system that utilized the global navigation satellite system (GNSS) to define a secure working area for logging. This study measured the effects of gait speed, transmission interval, geofence radius, and crossing angle information on the work environment for SA [29].

In the case of IoT devices, because the user’s information is collected through various sensors, it is possible to deduce the personal information of the user even if the collected data is captured. This paper analyzes the threats that may arise through various sensors included in IoT devices and measures the damage caused by the leakage of personal information that may occur through this. The next section elaborates the risk measurement method for SA.

## 5. Risk Measurement of Information Leakage in IoT Environment from the Viewpoint of SA

**Figure 4:**
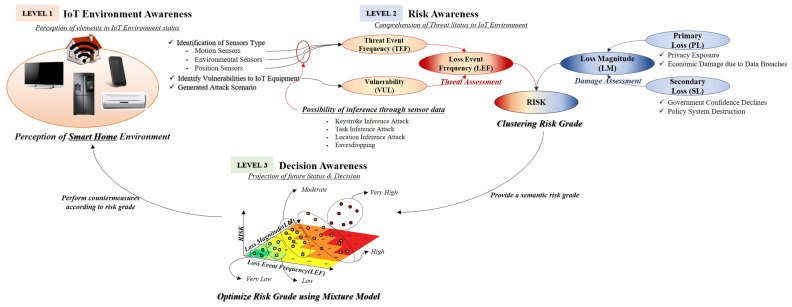
To make decisions about threat of mobile device’s sensors, SA proposed in this paper is composed of three levels: *IoT environment awareness* (*level 1*), *risk awareness* (*level 2*), and *decision awareness* (*level 3*).

Most risk analysis models use a qualitative method to measure the risk. However, it is not considered accurate owing to the disadvantage that these qualitative methods affect the degree of education, awareness, and experience of decision-makers. This section proposes a risk measurement method that combines clustering and optimization methods to minimize these qualitative judgments for risk. In addition, this study constructed a component of cyber SA in support of decision-making. The proposed system proposes *IoT environment awareness* (*level 1*) to identify threats and assets of IoT devices, *risk awareness* (*level 2*) to measure risks using the FAIR model, and *decision awareness* (*level 3*) that optimizes risk for decision support. This method adds the identification of IoT threat and loss of assets to our proposed threat measurement method (See Figure 4) [30,31].

### 5.1. IoT Environment Awareness For Identification of Threats and Assets

Most scope of assets present a range of information security management systems to meet the requirements of clause 5 of ISO 27001. This scope shall include interface management in accordance with the requirements. When setting the scope of an asset from the perspective of the information security management system (ISMS), it is important to identify the critical asset that can be exposed to the threat [32]. Identification of these assets is a starting point in the identification of vulnerabilities and threats in scenarios used to analyze risk. This level set the sensors used for IoT equipment to the range of assets.

Sensors are responsible for collecting data from objects. Unfortunately, if the software installed on the IoT device is infected with malware such as spyware, or if the user is exposed to Unsecured Wi-Fi, the IoT devices’ ID and password are exposed, The user can provide the attacker with a negative opportunity to access privacy data. The types of sensors installed in IoT devices are classified into motion sensors, environmental sensors, and position sensors. According to the research by A.K. Sikder et al., information leakage in IoT equipment is likely to be caused by light, motion, magnetic, acoustic, GPS, and camera sensors. The collected sensor data can expose passwords, life patterns, and personal location information [33] (see Table 1).

The attacker can deduce the propensity and privacy of the user through the collected data and leak personal information. These threats include keystroke inference attack, task inference attack, location inference attack, and eavesdropping.

- **Keystroke Inference Attack**: Keystroke inference is a common threat that can occur in IoT equipment. Most commercially available IoT devices comprise input devices such as touch screens, touch pads, and keyboards. When the user enters an ID, password, or word into the device, the device tilts and rotates to create a deviation of the data in the sensor (e.g., accelerometer, gyroscope, audio, light sensor, etc.) for each instance. An attacker can use this deviation of sensor data to infer a keystroke. Keystroke inference attacks can be made on IoT devices but they can also affect nearby devices. For example, putting the smartphone on a desk with a keyboard.
- **Task Inference Attack**: Task inference is a type of attack that infers information about an ongoing task or application on an IoT device. An attacker exploiting this inference attack can bypass the security policy implemented on the device and replicate the device state. The installed sensor in IoT device records the deviation of data values for various tasks being performed on the device. An attacker can use these values to infer execution processes and applications within the appliance.
- **Location Inference Attack**: The location inference is a location-privacy attack based on an acoustic side-channel. This inference attack utilizes acoustic information propagated in the nearest environment or space. This attack utilizes the acoustic reflection pattern of the voice at the user’s location and does not depend on the characteristic background noise. If the attacker can control IoT devices, they can identify personal information such as the user’s home or work location [34].
- **Eavesdropping**: IoT devices such as AI speakers use an audio sensor for dialing and receiving voice commands and other features. When a malicious application is installed on such IoT devices, it is possible to eavesdrop to record and extract the content and information of the conversation through such an audio sensor. Typical attacks that implement eavesdropping are Soundcomber and voice assistant applications. In this case, a malicious application uses an IoT device sensor to secretly record the user’s conversation [35]. Users are vulnerable to attack because the recording application runs in the background of the device. For example, when talking to a financial company such as a bank or a credit card company, a person may be exposed to threats if they reveal their personal information such as credit card or social security number. A malware that uses a voice assistant application can be used for a variety of malicious activities such as voice command duplication and information transmission. It can be controlled through SMS and Wi-Fi external control channel [36].

### 5.2. Risk Awareness to Set the Criteria for the Grades

Modern IT security focuses on measuring the risk to find a balance between opportunity realization and minimizing potential losses. From this perspective, *risk awareness* (*level2*) is the process of quantifying risk. The general formula used to measure risk is Risk=Threat×Vulnerability×Impact. However, this formula is a complete mathematical formula, instead of a model that demonstrates the concept of risk. To establish a complete mathematical formula, there should be a common and neutral measure of threats, vulnerabilities, and impact. However, they do not exist. This formula can be used conceptually because a relative portion of risk measurement depends on the nature of asset and the attacker. Threat×Vulnerability is generally used as the Likelihood value and can be matched with the *LEF* factors of the FAIR model. The threat is associated with the *TEF* factor of FAIR, including the attacker’s skill level, motivation, opportunity (the attacker possesses the necessary knowledge and access rights), and the attacker’s capabilities. Vulnerability is a variable that includes information on ease of discovery, ease of exploitation, awareness (known vulnerability), detection, and response, and is linked to a *VUL* factor including FAIR’s *TCap* and *CS*. Impact represents the damage to an asset due to a threat. These variables include the technical impact of data confidentiality, integrity, availability, and accountability, as well as business impacts such as financial and legal damages. It is linked to the LM, which represents the loss of assets in the FAIR (See Equation (1)).

(1) $$Risk=\underset{Likelihood}{\underbrace{\underset{\underset{TEF=Contact\times Action}{⏟}}{Threat}\times \underset{\underset{VUL=TCap\times CS}{⏟}}{Vulnerability}}}\times \underset{Loss\;Magnitude(LM)}{\underbrace{Impact}}$$

Existing risk results are generalized from “very high” to “very low”. However, because the correspondence varies according to the criteria of the grade, an error may occur in qualitative judgment according to the decision-maker. To overcome these limitations, the *risk awareness* level proposes a method to determine the criteria of the grade through clustering instead of classifying the measured risk value according to the specified grade criterion. The grade is determined by the risk value because assets, threats, etc. are relative to the structure or environment of the organization. Assuming that there are number of *n* sets of risk values $\left(risk_{1},risk_{2},\cdots ,risk_{n}\right)$, the risk grade $\left(G=\left\{G_{1},G_{2},...,G_{k}\right\}\right)$ divides into number of *k* grades that maximize the density between the risk values. In this case, if the set of risks belonging to the risk grade is $G_{i}$, and $mu_{i}$ is the mean of $G_{i}$, the variance $Var_{Risk}$ of risk can be expressed as Equation (2).

(2) $$Var_{Risk}=\sum_{i=1}^{k}\sum_{j\in G_{i}}{|risk_{j}-\mu_{i}|}^{2}$$

To find $G_{i}$ that minimizes $V_{Risk}$, *risk grade setting* and *risk grade rebalancing* steps are iteratively calculated until they converge (See Equations (3) and (4)) [37].

**Step** **1.** ***Risk Grade Setting**: The Euclidean distance of $\mu_{i}$ for each risk grade is calculated from each risk value and the risk is assigned by finding the risk grade closest to the risk value.*

(3) $$G_{i}^{\left(t\right)}=\{risk_{p}:|risk_{p}-\mu_{i}^{\left(t\right)}{|}^{2}\leq |risk_{p}-\mu_{j}^{\left(t\right)}{|}^{2}\;\forall j,1\leq j\leq k\}$$

**Step** **2.** ***Risk Grade Rebalancing**:* $\mu_{i}$ *is reset to the mean value of the risk values assigned to each risk grade.*

(4) $$\mu_{i}^{\left(t+1\right)}=\frac{1}{|G_{i}^{\left(t\right)}|}\sum_{risk_{j}\in G_{i}^{\left(t\right)}}risk_{j}$$

### 5.3. Decision Awareness for Optimization

Although clustering the measured risk and setting the grading criterion is not optimal, it may result in a critical point that should be qualitatively judged. The *decision awareness* level optimizes the assumption that the clustered risk grade is combined into *k* Gaussian distributions to compensate for these limitations. This optimization method is called mixture model. It is a method to statistically deduce the characteristics of the subgroups combined into *k* Gaussian distributions, implying that *k* risk grades set at *risk awareness* level are set to *k* Gaussian distributions at *decision awareness* level. The risk grade is expressed as $G_{k}=N\left(risk|\mu_{k},\Sigma_{k}\right)$ at this level.

Using the mixture model, $risk_{n}$ is set to be included in the risk grade clusters consisting of *k* Gaussian distributions and expressed as R. $z_{nk}$ is a binary variable that represents a value of 1 if the $risk_{n}$ belongs to the *k* th Gaussian distribution, and 0 otherwise. To optimize the risk grade, when given $risk_{n}$, a Gaussian distribution that maximizes R should be chosen. If the parameter of the *k* th Gaussian distribution for the risk grade is $\pi_{k}$, $\mu_{k}$, $\Sigma_{k}$, R can be expressed through Bayes’ theorem (See Equation (5)). In this case, $\pi_{k}=p\left(z_{nk}=1\right)$ means that $\pi_{k}$ and $p(z_{nk}=1)$ is selected for the *k* th risk grade.

(5) $$R\left(z_{nk}\right)=p\left(z_{nk}=1|risk_{n}\right)=\frac{p\left(z_{nk}=1\right)p\left(risk_{n}|z_{nk}=1\right)}{\sum_{j=1}^{K}p\left(z_{nj}=1\right)p\left(risk_{n}|z_{nj}=1\right)}=\frac{\pi_{k}N\left(risk_{n}|\mu_{k},\Sigma_{k}\right)}{\sum_{j=1}^{K}\pi_{j}N\left(risk_{n}|\mu_{j},\Sigma_{j}\right)}$$

In general, a mixture model uses an expectation-maximization (EM) algorithm for parameter estimation. To use the EM algorithm, Equation (5) is converted to a log likelihood function to calculate a value that optimizes the risk grade. The maximization method utilizes an alternative update that partially differentiates each π, μ, and Σ (See Equations (6)–(8)) [38,39].

(6) $$\begin{array}{ccc} \mu_{k} & = & \frac{\sum_{n=1}^{N}R\left(z_{nk}\right)risk_{n}}{\sum_{n=1}^{N}R\left(z_{nk}\right)} \end{array}$$

(7) $$\begin{array}{ccc} \Sigma_{k} & = & \frac{\sum_{n=1}^{N}R\left(z_{nk}\right)\left(risk_{n}-\mu_{k}\right){\left(risk_{n}-\mu_{k}\right)}^{T}}{\sum_{n=1}^{N}R\left(z_{nk}\right)} \end{array}$$

(8) $$\begin{array}{ccc} \pi_{k} & = & \frac{\sum_{n=1}^{N}R\left(z_{nk}\right)}{N} \end{array}$$

## 6. Results

This section measures the security risks that can occur in a smart home using the FAIR method. Using the FAIR method for risk measurement is not a new approach, but it contributes in terms of classifying the scope of risk by considering the environment for each scenario in the method of measurement. The results of this study measure the risk of possible scenarios based on security threats and assets that can be identified in the IoT based smart home environment. In addition, as IoT technology becomes more user-friendly, it also considers secondary damage due to information leakage.

### 6.1. Identify Asset and Threat in Smart Home

This study assumes that an attacker has the intention of taking data collected from sensors of IoT devices using malicious applications. This malicious application is divided into two types. The first is a type that replaces frequently used apps with a real app and second is a type that operates in the background of a device to steal internal data. When this malicious application is installed in IoT devices, it gets access to various sensors. This enables the unauthorised collection of information and monitoring of privacy. In order to utilize the risk assessment approach, it is necessary to set a range of information assets and to profile them. In this profiling process, the security requirements of the asset are identified. This determines where all the assets are stored, transported and processed. A user or system in a smart home identifies vulnerable points that can compromise security requirements for confidentiality, integrity, and availability, depending on how the asset is accessed. These identified information assets make it possible to identify security threats. The security vulnerabilities or the patches of concern are created as scenarios in connection with the threat attributes. This can identify specific threats that could have a negative impact on assets. This paper can use this scenario generation procedure to understand the impact of the smart home system and identify cyber and physical security risks in terms of information assets. In this study, the range of information assets at risk of possible outflows in the smart home was referenced by B. Ali and A. Awad [8].

The attacker imitates the attack as a user with legitimate rights. In order for the attacker to achieve the goal, the credentials of the smart home user are required. To do this, the user ID and password must be acquired. Access to credentials can be performed using social engineering techniques or by intercepting generic data collected through IoT sensors. Since the general data utilizes motion sensors and audio sensors, the collected data itself can be a weak point because it allows direct analysis of the data (*Scenario 1*) [40]. By injecting malicious code into the mobile application connected to the IoT system, an attacker can provide an opportunity to perform harmful operations. The malicious code insertion threat can cause damage due to leakage of general data through GPS sensors, motion sensors, audio sensors, and camera sensors mounted on a mobile device. In particular, such a threat could be utilized as an attack path for acquiring a user’s credentials (*Scenario 2*) [41,42]. Environmental sensors installed in the IoT device have the purpose of detecting a physical threat such as an abnormality in the home and warning the user of the danger. An attacker can steal information gathered from installed sensors by releasing the malicious code used in *Scenario 2* to the devices. This behavior can be used as an unauthorised surveillance tool to determine where the user is located in the home (*Scenario 3*) [43].

Through *Scenario 1*, an attacker can access the inventory information embedded in the IoT device and search for a specific device with a known vulnerability. Through this process, the attacker can not only identify the internal structure of the smart home but also can be used as a path to provide an attack on *Scenario 3* (*Scenario 4*) [44]. In particular, smart TVs that have been sold recently have built-in camera sensors. Also, privacy information leaked through the IP camera. If an attacker can identify IoT devices known as vulnerabilities (*Scenario 4*), an attacker can control camera sensors to monitor and spy on smart home users (*Scenario 5*) [45,46]. When an attacker accesses location data for a mobile device or a GPS-enabled device (*Scenario 1, 2*), it can conclude whether a smart home resident is present at home. This can lead to financial losses such as home robbery (*Scenario 6*) [47] (See Table 2).

IoT devices that are the basis of smart homes recognize human actions by mounting sensors. Sensors that recognize human actions are classified into motion sensors, environmental sensors, and position sensors. The data from these sensors occurs when an attacker unauthorisedly gains root privileges. The associated vulnerability is defined as a common weakness enumeration (CWE) 264. “CWE 264” is defined as *“weaknesses in this category related to the management of permissions, privileges, and other security features that are used to perform access control”* [48]. When an attacker gains permission or access to IoT devices, various sensor data stored in the device can be collected and analyzed to make inference about personal information. There are four attack methods such as keystroke inference, task inference, location inference, and eavesdropping.

**Figure 5:**
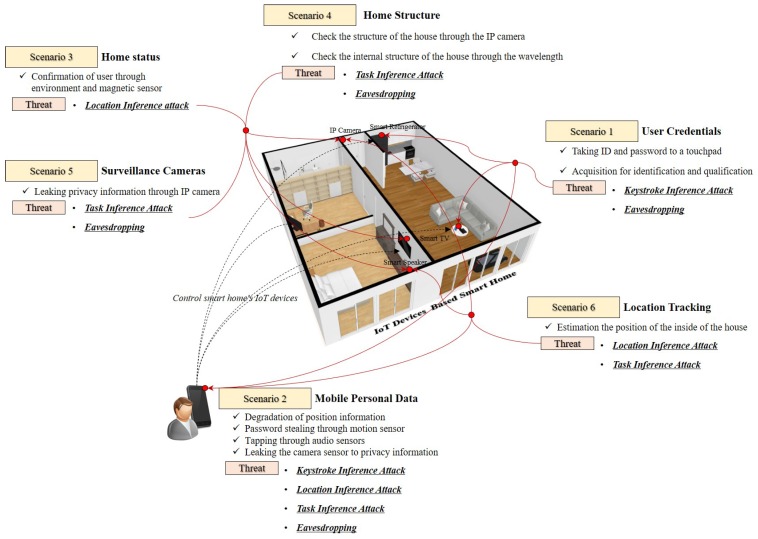
Sensors in IoT devices installed in smart homes can be used as information leakage path.

Threats to information assets in smart homes are linked to various losses. In particular, leakage of personal information through data breach can lead to economic losses. According to Strategy Analytics, the market outlook for smart homes is expected to reach a market value of $111.5 billion by 2019, compared with $57.5 billion in 2015. In Staitista, smart homes are expected to increase five times in 2020 compared to 2015 in the United States. However, contrary to this, security policy and legal regulations are insufficient. In this situation, when information is leaked by a smart home, the criticism can lead to national policy, which can lead to social confusion (see Figure 5).

### 6.2. Risk Measurement for Decision Making on IoT-based Smart Home

Applying a qualitative risk measurement model could lead to uncertain decisions in the process of SA. However, due to the nature of IoT equipment, attacks occur in various layers and decision-makers must consider the damage that extends from cyberspace to the real world. To address this, sophisticated risk measures that reflect the capacity and likelihood of threats, and the major and minor losses of assets are required.

#### 6.2.1. Threat Measurement for Smart Home

Threats are measured by likelihood, capacity, and vulnerability. In this regard, FAIR’s *LEF* is a set of factors that can make sophisticated measurements of threats. These LEFs are measured by the combination of frequency of occurrence of threats (*TEF*) and vulnerabilities (*VUL*) available to the threat agent.

- ***Threat Event Frequency* (*TEF*) for IoT Devices**: TEF is measured by a combination of *contact* and *action*. *Contact* is the frequency with which the threat agent tries to access the asset to be attacked and *action* is the possibility that the threat agent performs an attack against the attacked object. This study assumes that the contact has occurred because it uses the attack method to deduce the personal information by collecting data about the sensors included in the device. In other words, because there is a limit to obtain permission for various IoT devices, this study reflected the average of the accuracy of other studies when the inference attack was successful (see Table 3). The threat to *Scenario 1* is the keystroke inference and eavesdropping that can occur with motion sensors (accelerometer, gravity, gyroscope, and audio sensors.) The threat of *Scenario 2* is location inference and eavesdropping, so GPS sensor, motion sensors, It is based on the probability of inference. *Scenario 3* occurs at the location reference, which is exposed to threats through environmental sensors and magnetic sensors. *Scenario 4* is a threat to understanding the structure of a home, so eavesdropping and task inference attacks are used. This can be represented by a combination of the accuracy of the information inference of camera sensors, light sensors, and audio sensors. Since *Scenario 5* is an unauthorised surveillance threat, threats can be quantified by the probability of inference through camera sensors. *Scenario 6* is the combination of the inference probabilities of the position sensors with the threat of confirming whether the resident’s home location is located by location inference and task inference.

- ***Vulnerabilities* (*VUL*) of IoT Devices**: *VUL* is measured as a combination of *threat capability* (*TCap*) and *control strength* (*CS*), which indicates difficulty in successful attacks. Given the collection of sensor data and information leakage, it should reflect the vulnerability defined as “CWE 264” implying “gain privilege” [64]. Therefore, this study utilized the common vulnerability scoring system (CVSS) because it can generalize scores for vulnerabilities across software and hardware platforms and enable uniform vulnerability management policies. In addition, the CVSS score can be represented by the *TCap* of the FAIR because the attack vector, confidentiality impact, integrity impact, availability impact, exploit code maturity, and remediation level are considered in the process of calculating the lower equation. Because *CS* is a factor indicating the difficulty of attack, it is set to the price of the exploit traded in “Zerodium”. The reason for selecting *CS* for this price is that the higher the price of the exploit, the more difficult it is to find and use the vulnerability. In this study, *CS* was set as an index from 0 to 10 using the prices for exploits that can collect sensor data among transaction items (See Table 4) [65]. The vulnerabilities for each scenario are equally reflected by the combination of the CVSS Score defined as “CWE 264” and the exploit indicators traded at Zerodium. The reason for this is that we have exploited the vulnerability of privilege taking over IoT devices in terms of the probability distribution.

#### 6.2.2. Damage Measurement to Assets of Smart Home

*Loss magnitude* (*LM*) is a factor that measures the damage by an attack on the asset. This factor consists of *primary loss* (*PL*) and *secondary loss* (*SL*). *PL* is a factor that measures the direct damage caused by a threat agent and represents the economic damage suffered by an organization that owns the asset. This direct economic damage is related to productivity, restoration, and economic cost of information leakage. *SL* is secondary damage that can be caused by a threat agent. Most of the measures of secondary damage include additional economic damage due to legal liability or negative image formation on public opinion.

**Figure 6:**
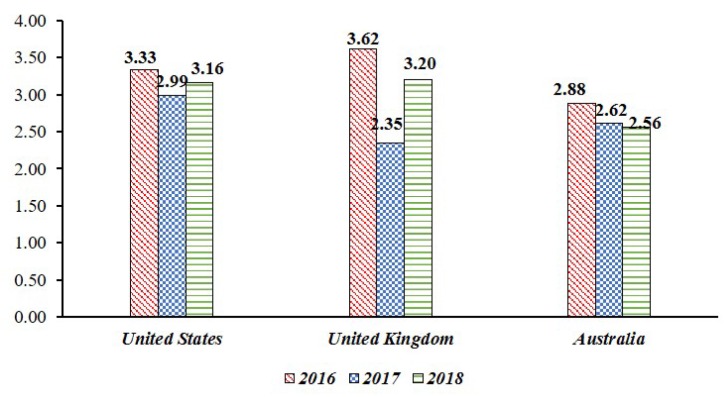
The *Primary Loss* (*PL*) score is based on the “Breach Level Index” data from 2016 to 2018, and the score is expressed as 0 to 10, but the risk for average data leakage is decreasing.

- ***Primary Loss* (*PL*) to Data Breach in Smart Home**: This paper assumes the leakage of sensor data collected from IoT devices in a smart home. In this case, the threat competence constituting the PLEF is the number of leaked data, and PLM is a combination of criticality, cost, and sensitivity, which is a direct economic loss caused by the threat. This PL data is equal to the risk level measured in the “Breach Level Index”. This study collected the data of “Other”, which represents the outflow of general information in the data breach, and used it as an indicator of PL. From 2016 to 2018, we used risk data for information leakage from three countries (United States, United Kingdom, Australia) that suffered the most data leakage (See Figure 6) [66].
- ***Secondary Loss* (*SL*) against Social Disruption**: In most cases of information leaks, organizations responsible for information management are subject to legal liability or public criticism. However, this legal responsibility and the level of public criticism are linked to whether the policy system is structured systematically. In other words, for information leakage events, government functionality, civil liberty, and political participation can be used as factors affecting secondary loss. Since *SLEF* considers timing, due diligence, response, and detection, it relates to the government functionalities corresponding to information leakage. With the case of *SLM*, it is associated with civil liberty and political participation in terms of including changes to the legal and regulatory landscape, competitive landscape, media, and external stakeholder. *SLM* was used as a civil liberty and political participation because it could suppress social confusion such as political participation or criticism of public opinion and public opinion. This study used government functionality, civil liberty and political participation among the national democracy indexes published in the “Economist” in setting SL (See Table 5) [67].

#### 6.2.3. Risk Optimization for Decision-Making

**Figure 7:**
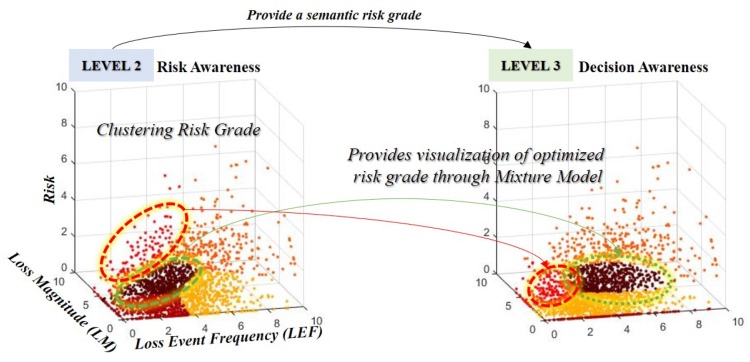
Clustering and optimization methods were combined with risk measurement and situational model to provide semantic visualization of risk grade.

The *TEF* generates and sets the Gaussian distribution based on the probability of information that can be inferred through the sensor. This indicates that the TEF of user credentials, which is an attackable scenario of the information asset, is generated by combining the probabilities of the threats of the corresponding threats. In addition, we combined other factors of FAIR to derive the risk of information leakage scenario through sensors. However, there is a relative aspect and a qualitative aspect of the classification of the risk of measuring the risk. To overcome this, the study clustered the risk grades from 0 to 10 into five ranges (*very low, low, moderate, high, very high*). Because this combination is complex, the proposed model adds a process to optimize the calculated risk (see Figure 7).

**Figure 8:**
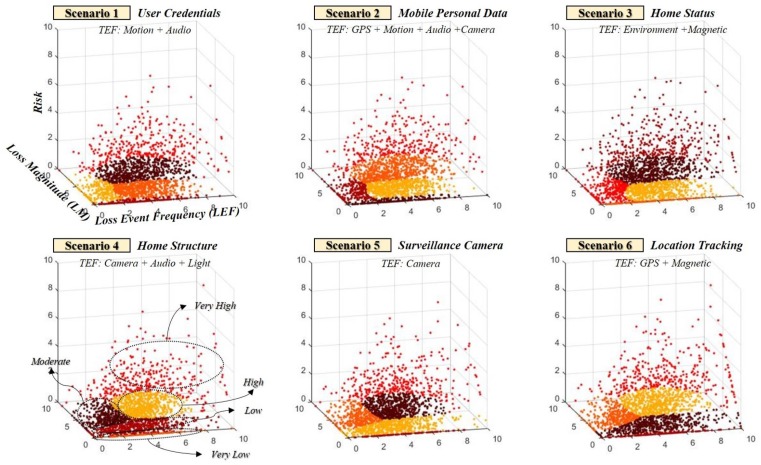
Relativity should be considered because criteria for risk grade range may change depending on the scenario.

**Figure 9:**
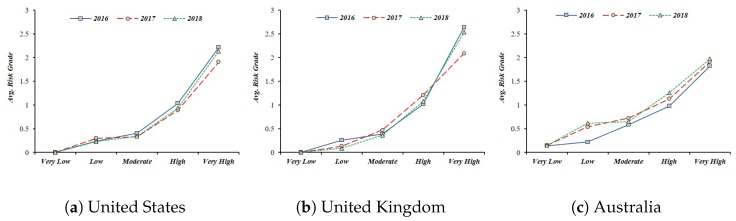
The average of the risk grade for *Scenario 1* is relative to the policies and circumstances of each country.

Risk criteria is relatively different according to each scenario occurring in a smart home, implying that even if the same five risk grades(*very low, low, moderate, high, very high*) are selected, they can change depending on the attack target, attack methods, and inference probability. This view means that the relativity of the risk can be changed considering the secondary damages to individuals, society, and the state. Because the value of private property such as a smart home differs for each user, it is necessary to consider the relative criteria of the situation rather than the uniform criteria for the risk grade (see Figure 8 and Figure 9).

## 7. Conclusions

With the increasing popularity of IoT devices, human living environments such as smart homes are being developed. This advancement creates a negative effect wherein users of IoT technology are vulnerable to attack. Especially, IoT devices installed in a smart home are equipped with various sensors. Therefore, the possibility of leakage of personal and private information (e.g., home condition, house structure, user’s taste, etc.) increases. Such information leakage is economically damaging to individuals and causes social and political damage. Hillary Clinton’s e-mail leak, which occurred during the US presidential election in 2016, was also at home, a private space, and a threat to smart homes is at the starting point. Therefore, inference attack research using several sensor data is underway.

However, these researches focus on detection instead of the measurement of risk. This study presented an attack scenario on the assumption that sensor data was leaked through the vulnerability of smart home IoT equipment. This paper suggests the risk measurement method and risk grade classification through FAIR and clustering method based on the scenario. In addition, a mixture model is proposed to express semantic results in terms of SA. If existing risk measurement methods have limitations in terms of utilizing qualitative, heuristic, and empirical aspects, this study enabled quantitative risk measurement considering the relativity of risk assessment. In the case of a smart home, IoT equipment is configured and utilized differently for each user. Therefore, relativity and quantification are required to cope with the risk. The proposed method provides decision-makers with a semantic perspective on the threats of various IoT devices and derives the quantitative risk of the damages caused by them. It also quantifies the legal remedy of the leakage of personal information of IoT equipment in the future.

From the perspective of situational awareness, risk assessment is aimed at inducing decision makers to make appropriate decisions. However, existing qualitative risk assessments tend to update on risk indicators once determined because they are more reflective of cognitive and empirical aspects of security experts than data-based ones. Because the proposed model utilizes data-based measurement results, it can acquire a convergence risk rating for each security scenario with continuous data updates. In addition, risk indicators can be set so that various decision makers can adapt to the resources held by their business environment in that they can be grouped into the range of risk grades they want. The results of this paper confirm that the risk distribution can change with each scenario, country and time. However, actual security sites are preparing for threats by deriving a uniformized risk grade. A typical example is the Information Operations Condition (INFOCON) grade. This study will provide a basis for a real-time response to the rapidly changing security operating environment of the future. It can also contribute to periodic risk assessment in response to cyber threats, cyber attacks and cyber warfare from a perspective of national security. 

References

## Figures and Tables

**Table 1:** Mobile device sensors can be classified into motion sensors, environmental sensors, and position sensors. Information leakage is possible by integrating data from these sensors [33].

Sensor Type	Sensor	Description
Motion Sensors	Accelerometer	- Measure acceleration along the X, Y, and Z axes - Possible to check the speed change or strength of the mobile device
	Gravity	- Measures gravitational acceleration based on the X, Y, and Z-axes - Recognizes the horizontal or vertical direction and the upper and lower references
	Gyroscope	- Measures the rotational speed for the X, Y, and Z-axes - Checks mobile device tilt or rotation
Environmental Sensors	Light sensor	- Measures light in lx - Used to adjust the screen brightness of the mobile device depending on the surrounding environment
	Temperature sensor	- Measures the ambient temperature - Sets or controls the temperature of the mobile device
	Proximity sensor	- Measures the distance between the screen of the mobile device and the object to be measured without physical contact
	Audio sensor	- Microphone: Detects acoustic signal - Speaker: Plays audio signal
	Camera sensor	- Handles lighting intensity and ambiance for capturing photos and videos around mobile devices
	Barometer sensor	- Pressure measurement of mobile device
Position Sensors	GPS sensor	- Uses GPS satellites to measure the current location and time of the mobile device
	Magnetic sensor	- Measures the azimuth using the Earth’s magnetic field, and applies it to compass applications

**Table 2:** Threats and attack methods that can occur according to information assets that create various scenarios.

Scenario	Asset	Possible Threats	Attack Methods
1	User credentials	- Taking ID and password to a touchpad using a motion sensor - Acquisition of audio sensor data for identification and qualification	-Keystroke Inference -Eavesdropping
2	Mobile personal data	- Degradation of position information through GPS sensor - Password stealing through motion sensor - Tapping through audio sensors - Leaking the camera sensor to privacy information	-Keystroke Inference -Location Inference -Task Inference -Eavesdropping
3	Home status	- Confirmation of user through environment sensor and magnetic sensor	-Location Inference
4	Home structure	- Check the structure of the house through the camera sensor - Check the internal structure of the house through the wavelength recognized by the audio and light sensor	-Eavesdropping -Task Inference
5	Surveillance cameras	- Leaking privacy information through camera sensor	-Eavesdropping -Task Inference
6	Location tracking	- Using the GPS sensor to estimate the position of the inside of the house - Using the magnetic sensor to estimate the position of the inside of the house	-Location Inference -Task Inference

**Table 3:** This study sets TEF index as attack potential when an inference attack is performed through data collected by various sensors attached to IoT equipment.

Type of Sensor	Attackable Sensors	Possibility of Attack (Index of *TEF*)	Ref.
Motion Sensors	Accelerometer, Gravity Gyroscope	0.762 (TEF: 7.62)	[49,50,51,52,53]
Environmental Sensors	Light sensor	0.825 (TEF: 8.25)	[54,55]
	Audio sensor	0.805 (TEF: 8.05)	[36,56,57,58]
	Camera sensor	0.79 (TEF: 7.9)	[59,60,61]
Position Sensors	GPS sensor	0.869 (TEF: 8.69)	[34]
	Magnetic sensor	0.96 (TEF: 9.6)	[62,63]

**Table 4:** During Zerodium’s vulnerabilities trade, this study generated price index based on remote code execution (RCE) and local privilege escalation (LPE) vulnerabilities associated with permissions.

Price	Vulnerabilities	Index of *Control Strength* (*CS*)
Up to $500,000	Email App RCE + LPE	9.5
	SMS/MMS RCE + LPE	9.18
	Signal RCE + LPE	8.22
	Viber RCE + LPE	7.58
Up to $1,500,00	Chrome RCE + LPE	6.94
	Documents RCE + LPE	6.62
	Media Files RCE + LPE	6.3
	Baseband RCE + LPE	5.98
Up to $1,000,00	LPE to Kernel	5.34
	Wifi RCE + LPE	4.7
Up to $50,000	LPE to Root	4.04
	RCE via MitM	3.72
Up to $25,000	LPE to System	1.5
Up to $15,000	Information Disclosure	0.32

**Table 5:** The *Secondary Loss* (*SL*) Score is the average of government functionality, civil liberties, and political participation.

Year	Nations	Government Functionality	Political Participation	Civil Liberty	*Secondary Loss* (*SL*) Score
2016	United States	7.14	8.13	8.24	7.84
	United Kingdom	7.14	8.75	9.12	8.34
	Australia	8.93	7.78	10.0	8.90
2017	United States	7.14	7.22	8.24	7.53
	United Kingdom	7.50	8.33	9.12	8.32
	Australia	8.93	7.78	10.0	8.90
2018	United States	7.14	7.78	8.24	7.72
	United Kingdom	7.14	8.75	9.12	8.32
	Australia	8.93	7.78	10.0	8.90
